# Visual coronary artery calcification score to predict significant coronary artery stenosis in patients presenting with cardiac arrest without ST-segment elevation myocardial infarction

**DOI:** 10.1186/s13613-025-01423-5

**Published:** 2025-04-07

**Authors:** Maxence Brunel, Brahim Harbaoui, Laurent Bitker, Carole Chambonnet, Matthieu Aubry, Loïc Boussel, Cyril Besnard, Jean-Christophe Richard, Pierre Lantelme, Pierre-Yves Courand

**Affiliations:** 1 Fédération de cardiologie, Hôpital de la Croix-Rousse et Hôpital Lyon Sud, Hospices Civils de Lyon, 103 Grande Rue de la Croix-Rousse, Lyon, F-69004 France; 2 Université de Lyon, CREATIS; CNRS UMR5220; INSERM U1044; INSA-Lyon; Université Claude Bernard Lyon 1, Lyon, France; 3 Service de médecine intensive - réanimation, Hôpital de la Croix-Rousse, Hospices Civils de Lyon, Lyon, France; 4 Service d’imagerie médicale, Hôpital de la Croix-Rousse, Hospices Civils de Lyon, Lyon, France; 5 Service de cardiologie, Hôpital Nord-Ouest, Villefranche sur Saône, France

**Keywords:** Cardiac arrest, Sudden cardiac death, Ventricular fibrillation, Myocardial infarction, Coronary artery calcification

## Abstract

**Background:**

Emergency coronary angiogram after a cardiac arrest without ST-segment elevation myocardial infarction (STEMI) is still a matter of debate. To better select patients who may benefit from this procedure, we tested a visual coronary artery calcification (VCAC) score available in chest CT to predict significant coronary artery stenosis and/or culprit lesion or ad hoc or delayed percutaneous coronary intervention (PCI).

**Results:**

A total of 113 patients with cardiac arrest and without STEMI who had a coronary angiogram and chest CT (January 2013 to March 2023, Croix-Rousse Hospital, Lyon, France) were retrospectively included. VCAC was scored from 0 (no calcification) to 3 (diffuse calcification) for each 4 four main arteries (left main, left anterior descending, circumflex, and right coronary artery). At baseline the median [interquartile range] age was 65.8 years [53.4–75.7], 61.9% were male, and 59.3% presented with ventricular fibrillation. Coronary angiogram identified at least one significant coronary artery stenosis in 32.7%, and ad hoc and delayed PCI were performed in 12.4% and 6.2% of the patients, respectively. VCAC score was an excellent predictor of significant coronary artery stenosis with an area under the ROC curve (AUC) of 0.95 (95%CI [0.90-1.00]) and the optimal threshold was ≥ 4 (specificity 94.7%, sensitivity 91.9%). For the detection of culprit coronary artery stenosis, the AUC was at 0.90 (95%CI [0.85–0.96]) and the optimal threshold was ≥ 5 (specificity 83.5%, sensitivity 87.5%). The AUC was 0.886 [0.823–0.948] (specificity 81.8%, sensitivity 85.7%) for ad hoc PCI and 0.921 [0.872–0.972] (specificity 85.3%, sensitivity 88.9%) for both delayed and ad hoc PCI with a same optimal threshold of VCAC ≥ 5. A VCAC score ≥ 4 had a sensitivity at 100% to predict a significant or culprit coronary artery stenosis and ad hoc or delayed PCI.

**Conclusions:**

The present study found that a non-dedicated CT thorax may be useful to measure VCAC and if this is scored ≥ 4 it allows physicians to better select patients resuscitated from cardiac arrest with non-STEMI and without history of coronary artery disease who may benefit from an emergency coronary angiogram to detect a significant or culprit coronary artery stenosis and had PCI if appropriate.

**Supplementary Information:**

The online version contains supplementary material available at 10.1186/s13613-025-01423-5.

## Background

The burden of sudden cardiac death (SCD) worldwide is currently 4–5 million cases every year [1]. Even among those who undergo successful resuscitation and admission to the hospital, the prognosis of patients who have out-of-hospital cardiac arrest remains poor; the mortality rate is around 65% [2. Nearly 60% of out-of-hospital cardiac arrests are related to acute coronary syndromes [3]. Additionally, population-based studies suggest that up to 40% of patients who have SCD have a myocardial infarction scar at autopsy without previous knowledge or diagnosis of the disease [4]. In clinical practice, guidelines recommend to perform routinely an emergency coronary angiogram in patients with ST-segment elevation on post resuscitation electrocardiography (ECG) because of its good positive predictive value for acute coronary lesions [5]. In patients with cardiac arrest who do not have ST-segment elevation on ECG, immediate coronary angiography is not recommended because only two-thirds of patients have a significant coronary artery stenosis and only a third have a culprit lesion in two recent randomised controlled trials [6, 7]. In this clinical setting, it would be appropriate to better select patients who may benefit from immediate coronary angiogram, a costly and invasive procedure with possible side effects [8]. Coronary artery calcification (CAC) may be observed with chest computed tomography (CT), which is a non-invasive procedure frequently requested in post cardiac arrest. Visualised CAC (VCAC) in patients undergoing CT screening for lung cancer has previously been shown predictive of death from cardiovascular disease [9, 10]. We postulated that VCAC on chest CT may be helpful in patients without ST-segment elevation after cardiac arrest to identify those who had a higher frequency of significant or culprit coronary artery lesion and may benefit from an emergency coronary angiogram.

The aim of the present study was to test VCAC, as previously described [9, 10], to predict the presence of a significant coronary artery stenosis diagnosed with a coronary angiogram (primary objective) and/or a culprit artery stenosis or a percutaneous coronary intervention (PCI; secondary objectives: ad hoc or ad hoc and delayed) in patients presenting with a cardiac arrest without ST-segment elevation myocardial infarction.

## Methods

### Population

Our usual management of cardiac arrest follows the current guidelines. At intensive care unit admission, a standardised diagnostic procedure was initiated, with immediate coronarography in patients with ST-segment elevation or with high probability of acute coronary occlusion, or delayed procedure in patient with suspected cardiac arrest of cardiac origin. Cerebral and thoracic computed tomography with injection of contrast agent, cardiac ultrasonography, and possibly blood and urine toxicological analysis by high performance liquid chromatography were performed in selected cases [11]. We conducted a retrospective cohort study at the Croix Rousse Hospital (Hospices Civils de Lyon, Lyon, France). The inclusion criteria were patients presenting with a cardiac arrest without ST-segment elevation myocardial infarction admitted for a coronary angiogram and having undergone a routine chest CT less than one year before or after the coronary angiogram. All patients with a coronary angiogram from January 1, 2013 to March 31, 2023, were retrospectively screened for eligibility. For patients with more than one coronary angiogram following cardiac arrest, only the first exam was considered in the analysis. Patients with history of PCI or coronary artery bypass grafting were excluded.

### Data collection

The following data were collected from electronic medical files: demographic characteristics (age, gender), comorbidities (hypertension, diabetes, dyslipidaemia, smoking status), clinical presentation at first medical contact (asystole, ventricular fibrillation), ECG and transthoracic echocardiography at admission and laboratory parameters (plasma troponin peak).

### Coronary angiogram and PCI analyses

Significant coronary artery stenosis was defined as a stenosis > 70% on the coronary angiogram; these retrospectively classified as a culprit coronary artery stenosis when the stenosis was > 90% or when a thrombotic aspect was observed. Intravascular coronary imaging was not routinely performed. Patients were classified into five subgroups: normal, atherosclerosis without significant stenosis, one-vessel disease, two-vessel disease, and three-vessel disease. Ad hoc PCI was defined as PCI performed immediately during the same procedure, and delayed PCI as PCI performed during another procedure (a few days or weeks later). Revascularisation was decided by the interventional cardiologist who observed a suspected coronary culprit lesion.

### Visual scale for coronary artery calcification scoring

CT acquisition was performed on CT scanners with at least a 4-cm z-coverage: Brilliance 64 and iCT (Philips, Best, the Netherlands) or Discovery CT750 HD (GE medical systems Waukesha, WI, US). We used the same method as reported by Shemesh et al. [10]. Interpretation was performed without cardiac gating (*N* = 113) and preferably without intravenous contrast in coronary artery (*N* = 111) to optimise visual interpretation. Each of the four main coronary arteries was identified (left main trunk, left anterior descending, circumflex, and right). Considering the CT imaging conducted the closest to the cardiac arrest, calcification in each artery was categorised as absent (0), mild (1; less than one-third of the length of the entire artery showing calcification), moderate (2; one-third to two-thirds of the artery showing calcification) and severe (3; more than two-thirds of the artery showing calcifications); each subject received a VCAC score ranging from 0 to 12. The analyses of all CT were blinded to the results of the coronary angiogram and the PCI decision using an access to imaging that did not contain other medical data. The inter-reader agreement for VCAC scoring was previously evaluated, and a high level of overall agreement found by trained radiologists [10]. Some examples of VCAC scoring are illustrated in supplementary data (Supplementary Figures S1, S2, and S3). In the present study the inter-reader reproducibility between two non-radiologist physicians (MB, resident in cardiology, and PYC, senior cardiologist) was tested. After an initial analysis of a sample of 20 randomly-selected chest CT considered for analysis in the present study, we observed a clinically significant difference of VCAC score between the two physicians (mean ± standard deviation, SD: 3.90 ± 2.86 vs. 6.05 ± 3.20, *p* < 0.001), with an intraclass correlation coefficient of 0.810 (95% confidence interval, CI [0.581–0.920], *p* < 0.001) and moderate agreement with a Kappa coefficient of 0.286 (VCAC < 4 vs. VCAC ≥ 4). During this training phase, calcifications of aortic valve and mitral annulus or pericardium were notably misinterpreted as coronary artery calcifications. After corrections of misinterpretations, a second sample of 20 randomly-selected chest CT were analysed and VCAC scores were very close without clinically significant difference (mean ± standard deviation, SD: 4.25 ± 2.85 vs. 4.75 ± 2.29, *p* = 0.066), with an intraclass correlation coefficient of 0.844 (95% confidence interval, CI [0.653–0.935], *p* < 0.001 and a good agreement with a Kappa coefficient of 0.886 (VCAC < 4 vs. VCAC ≥ 4). Several months later, all 113 chest CT were analysed by a second physician; the intraclass correlation coefficient was 0.955 (95% confidence interval, CI [0.936–0.969], *p* < 0.001 and a good agreement with a Kappa coefficient of 0.923 (VCAC < 4 vs. VCAC ≥ 4).

### Statistical analyses

Quantitative variables were summarised as mean ± SD, except those with skewed distributions using measured skewness (Kolmogorov-Smirnov test) that were expressed as medians [interquartile range, IQR]. Categorical variables were expressed as count (percentage). Non-parametric (Jonckheere-Terpstra) tests were used to compare continuous variables between terciles groups of VCAC (VCAC = 0; VCAC 1–3; VCAC ≥ 4) as appropriate. Pearson’s χ² -test was used to compare dichotomous variables.

To estimate the overall accuracy of VCAC score for the diagnosis significant coronary artery stenosis (primary objective) or culprit coronary artery stenosis or ad hoc or ad hoc and delayed PCI (secondary objectives) an empirical receiver-operating characteristic (ROC) curve was built. The area under the ROC curve (AUC) and its 95% CI were estimated using the Mann-Whitney statistic and was compared to 50%. Sensitivity and specificity at optimal threshold were defined using the Youden index. Data are presented after the interpretation of VCAC by the first physician and in the supplementary data after the interpretation of VCAC by the second physician. In addition, the threshold to rule out coronary artery stenoses and PCI, namely a negative predictive value of 100%, is also presented. A sensitivity analysis was performed after exclusion of patients who had a thorax CT performed before or after the index hospital stay. model. AUC were also presented for other variables with usual variables associated with coronary artery stenosis.

The analyses were performed using SPSS software, release 20.0.0 (SPSS, Chicago, IL, US). A *p* value **<** 0.05 was considered for statistical significance.

## Results

### Baseline characteristics

**Fig. 1:**
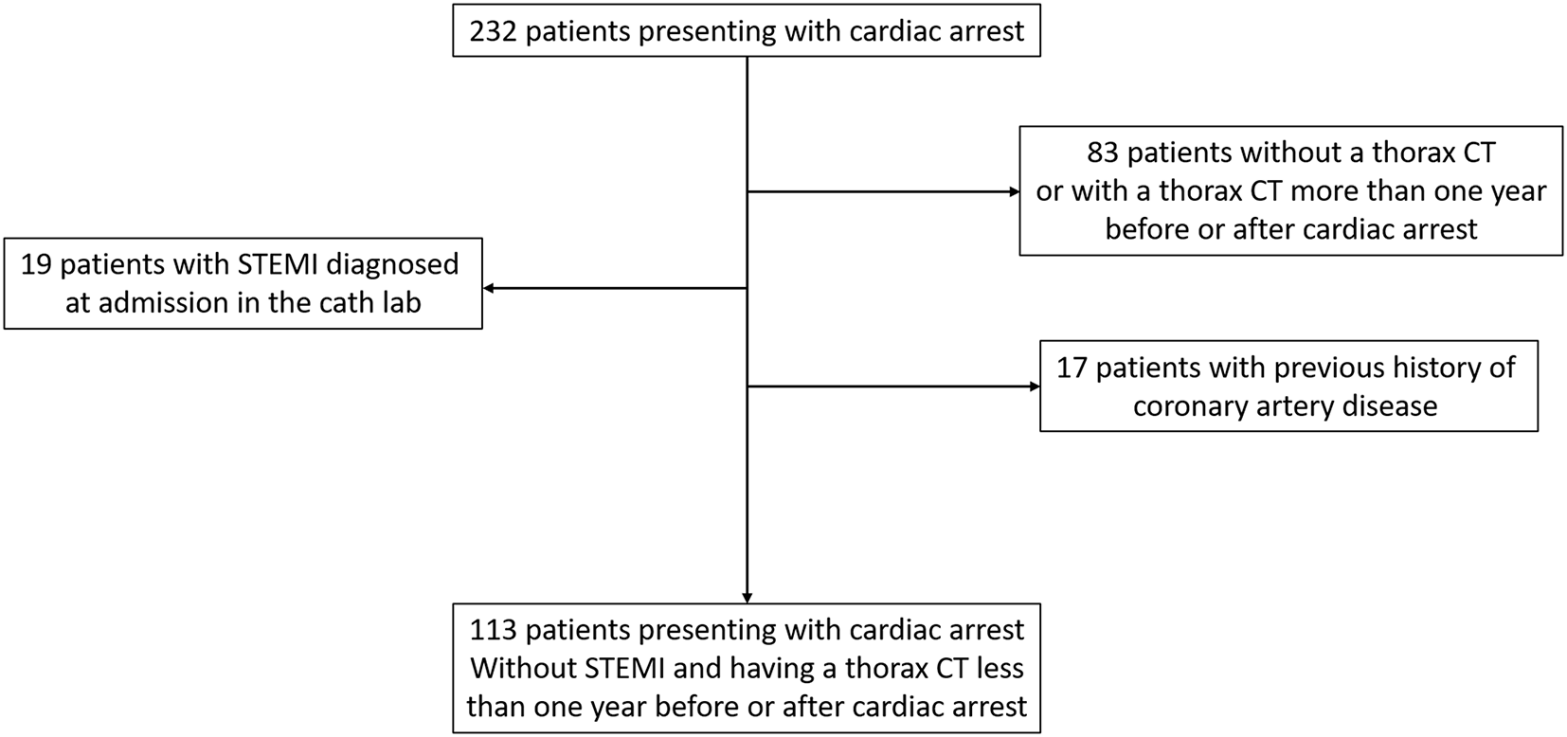
Study flowchart. CT: computed tomography; STEMI: ST elevation myocardial infarction

A total of 113 patients were included (Fig. 1). Seventy (61.9%) patients were men and the median age at admission was 65.8 years. Seventy-seven (68.1%) patients were admitted after out-of-hospital cardiac arrest. Initial rhythm was ventricular fibrillation in 67 (59.3%) patients. Patients with higher VCAC terciles were older and had higher BMI. They were more frequently had diabetes and hypertension. They received a lower dose of epinephrine and they were less frequently treated with prehospital extracorporeal life support (Table 1). Significant coronary artery stenoses were found during coronary angiogram in 37 (32.7%) patients: 21 (18.6%) had one-vessel disease, 10 (8.8%) had two-vessel disease, and 6 (5.3%) had three-vessel disease. Culprit coronary artery stenosis were observed in 16 patients: 15 had stenosis > 90% and 1 had an occlusion. Nine of them had a thrombotic aspect. None of them had coronary artery dissection or intramural haematoma. Ad hoc PCI was performed in 14 (12.4%) patients: 1 left main, 10 left anterior descending, 2 right coronary artery, and 1 circumflex. For ad hoc PCI, all stenoses treated were supposed as the culprit lesion: one artery was occluded, 12 had a stenosis > 90%, and one artery had a stenosis 70-89%. Nine of the 14 lesions had a thrombotic aspect at coronary angiogram. Delayed PCI was performed in 7 (6.2%) patients: 6 left anterior descending, and 1 right coronary artery (Table 2).

**Table 1:** Baseline characteristics of the total population and according to terciles of VCAC

	Total population (*N* = 113)	VCAC = 0 (*N* = 35)	VCAC 1–3 (*N* = 40)	VCAC ≥ 4 (*N* = 38)	*p*
Age (years)	65.8 [53.4–75.7]	52.9 [38.7–68.1]	62.7 [54.9–73.2]	75.1 [65.7–85.1]	< 0.001
Gender (male)	70 (61.9)	18 (51.4)	28 (70.0)	24 (63.2)	0.251
BMI (kg/m²)	27.7 [23.8–31.3]	27.3 [21.7–30.4]	27.6 [23.4–28.2]	31.5 [25.8–34.7]	0.048
Medical history					
Diabetes	27 (23.9)	3 (8.6)	12 (30.0)	12 (31.6)	0.037
Hypertension	47 (41.6)	8 (22.9)	15 (37.5)	24 (63.2)	0.002
Dyslipidaemia	25 (22.1)	3 (8.6)	11 (27.5)	11 (28.9)	0.066
Coronary family history	4 (4.7)	2 (5.7)	0 (0)	2 (5.3)	0.319
Tobacco never / former / current	59 (52.2) / 24 (21.2) / 30 (26.5)	19 (54.3) / 18 (45.0) / 22 (57.9)	4 (11.4) / 9 (22.5) / 11 (28.9)	22 (57.9) / 11 (28.9) / 5 (13.2)	0.124
Peripheral artery disease	7 (6.2)	1 (2.9)	2 (5.0)	4 (10.5)	0.369
Stroke	5 (4.4)	1 (2.9)	3 (7.5)	1 (2.6)	0.500
Atrial fibrillation	15 (13.3)	1 (2.9)	5 (12.5)	9 (23.7)	0.032
Cardiac arrest					
Out-of-hospital	77 (68.1)	26 (74.3)	28 (70.0)	23 (60.5)	0.430
Time from arrest to basic life support (min)	1 [0–5]	3 [0–5]	1 [0–4]	1 [0–5]	0.401
Time from arrest to return of spontaneous circulation (min)	23 [11–33]	27 [14–45]	20 [6–32]	22 [12–30]	0.174
Epinephrine dose during resuscitation (mg)	2 [1–4]	3 [1–5]	2 [1–3]	2 [0–4]	0.034
Ventricular fibrillation at baseline	67 (59.3)	15 (42.9)	15 (37.5)	16 (42.1)	0.874
Invasive mechanical ventilation	108 (95.6)	35 (100)	37 (92.5)	36 (94.7)	0.276
Targeted temperature management	90 (79.6)	29 (82.9)	32 (80.0)	29 (76.3)	0.784
Haemodynamic instability requiring inotropes or vasopressors drugs after cardiac arrest	41 (36.3)	15 (42.9)	13 (32.5)	13 (34.2)	0.615
Prehospital extracorporeal life support	7 (6.2)	5 (14.3)	2 (5.0)	0 (0)	0.038

The data are n (%) or median [interquartile range, IQR]

BMI: body mass index, VCAC: visual coronary artery calcification

**Table 2:** Laboratory and imaging characteristics of the total population and according to terciles of VCAC

	Total population (*N* = 113)	VCAC = 0 (*N* = 35)	VCAC 1–3 (*N* = 40)	VCAC ≥ 4 (*N* = 38)	*p*
ECG characteristics after resuscitation					
Normal	63 (55.8)	22 (62.9)	24 (60.0)	17 (44.7)	0.237
Atrial fibrillation	13 (11.5)	1 (2.9)	4 (10.0)	8 (21.1)	0.048
Left bundle branch block	11 (9.7)	2 (5.7)	6 (15.0)	3 (7.9)	0.358
Right bundle branch block	18 (15.9)	5 (14.3)	6 (15.0)	7 (18.4)	0.873
QT prolongation	3 (2.7)	2 (5.7)	0 (0)	1 (2.6)	0.307
Isolated abnormal T waves	5 (4.4)	3 (8.6)	0 (0)	2 (5.3)	0.188
Pacing	4 (3.5)	2 (5.7)	0 (0)	2 (5.3)	0.319
Laboratory parameters					
eGFR (mL/min)	67 [43–85]	68 [48–89]	65 [44–79]	65 [36–89]	0.274
Troponin at admission normalised to ULN	4.6 [1.3–21.5]	10.2 [1.5–85.9]	4.2 [0.8–19.9]	4.6 [1.3–13.2]	0.237
Peak troponin normalised to ULN	32.0 [4.1-174.7]	55.5 [4.6-297.9]	10.1 [3.8–93.5]	42.6 [10.8-190.3]	0.713
Transthoracic echocardiography					
LVEF (%)	50 [30–60]	50 [30–60]	50 [30–60]	40 [30–60]	0.528
Systolic function					0.514
Normal LVEF	50 (44.2)	16 (48.5)	19 (52.8)	15 (41.7)	
Diffuse hypokinesia	33 (29.2)	11 (33.3)	12 (33.3)	10 (27.8)	
Segmental hypokinesia	22 (19.5)	6 (18.2)	5 (13.9)	11 (30.6)	
Coronary artery calcium scoring					
Total	2 [0–5]	0 [0–0]	1 [1–2]	6 [5–8]	< 0.001
Left main	0 [0–1]	0 [0–0]	0 [0–0]	1 [1–2]	< 0.001
Left anterior descending	1 [0–2]	0 [0–0]	1 [1–1]	2 [2–3]	< 0.001
Circumflex	0 [0–1]	0 [0–0]	0 [0–0]	1 [1–1]	< 0.001
Right coronary artery	0 [0–1]	0 [0–0]	0 [0–1]	1 [1–2]	< 0.001
Coronary angiogram					< 0.001
Normal coronary angiogram (%)	42 (37.2)	26 (74.3)	16 (40.0)	0 (0)	
Atherosclerosis without significant coronary artery stenoses, n (%)	34 (30.1)	8 (22.9)	22 (55.0)	4 (10.5)	
1-vessel disease, n (%)	21 (18.6)	1 (2.9)	2 (5.0)	18 (47.4)	
2-vessel disease, n (%)	10 (8.8)	0 (0)	0 (0)	10 (26.3)	
3-vessel disease, n (%)	6 (5.3)	0 (0)	0 (0)	6 (15.8)	
Number of vessels diseased	0 [0–1]	0 [0–0]	0 [0–0]	1 [1–2]	< 0.001

The data are n (%) or median [interquartile range, IQR]

eGFR: estimated glomerular filtration rate; LVEF: left ventricular ejection fraction; ULN: upper limit of normal, VCAC: visual coronary artery calcification

At the end of hospital stay, suspected aetiologies of cardiac arrest were as follows: 41 primary cardiac arrythmia (36.3%) including ischaemic and non-ischaemic causes (myocardiopathy, channelopathy) but excluding metabolic disorder, 20 hypoxaemia related to heart failure (17.7%), 14 hypoxaemia related to pneumonia (12.4%), 11 of unknown origin (9.7%), 9 metabolic disorder (8.0%), 6 stroke (5.3%), 4 drug intoxication (3.5%), 3 pulmonary embolism (2.7%), 3 anaphylactic origin (2.7%), 1 traumatic context (0.9%), and 1 digestive cause (0.9%).

### Visual coronary artery calcium score

Chest CT scans were performed a median [IQR] 0 [0–1] days before or after coronary angiogram [72 (63.7%) the same day], and during the same hospital stay in 106 patients (93.8%). The median [IQR] VCAC score was 0 [0–1] for the left main, 1 [0–2] for the left anterior descending, 0 [0–1] for the circumflex, and 0 [0–1] for the right coronary artery. The median [IQR] total VCAC score was 2 [0–5]; 35 patients (31.0%) had a total VCAC score of 0. Individual data are plotted for each artery in and for total score in each patient in supplementary data (Figure S4 and S5, respectively).

### Relation between visual coronary artery calcium score and coronary angiogram

**Fig. 2:**
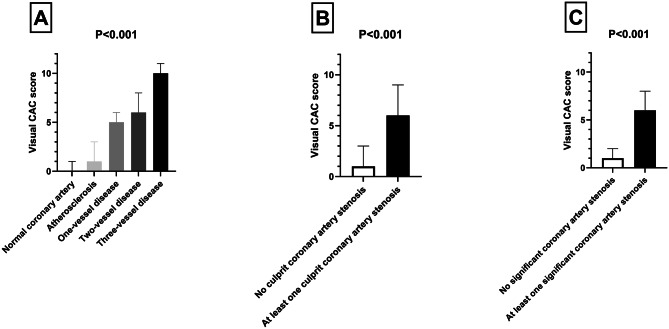
VCAC (visual coronary artery calcification) score according to the severity of coronary artery disease observed during the coronary angiogram. Data are median and interquartile range, *P* value using Jonckheere-Terpstra test for panel **A** and Mann-Whitney test for panel **B**

**Fig. 3:**
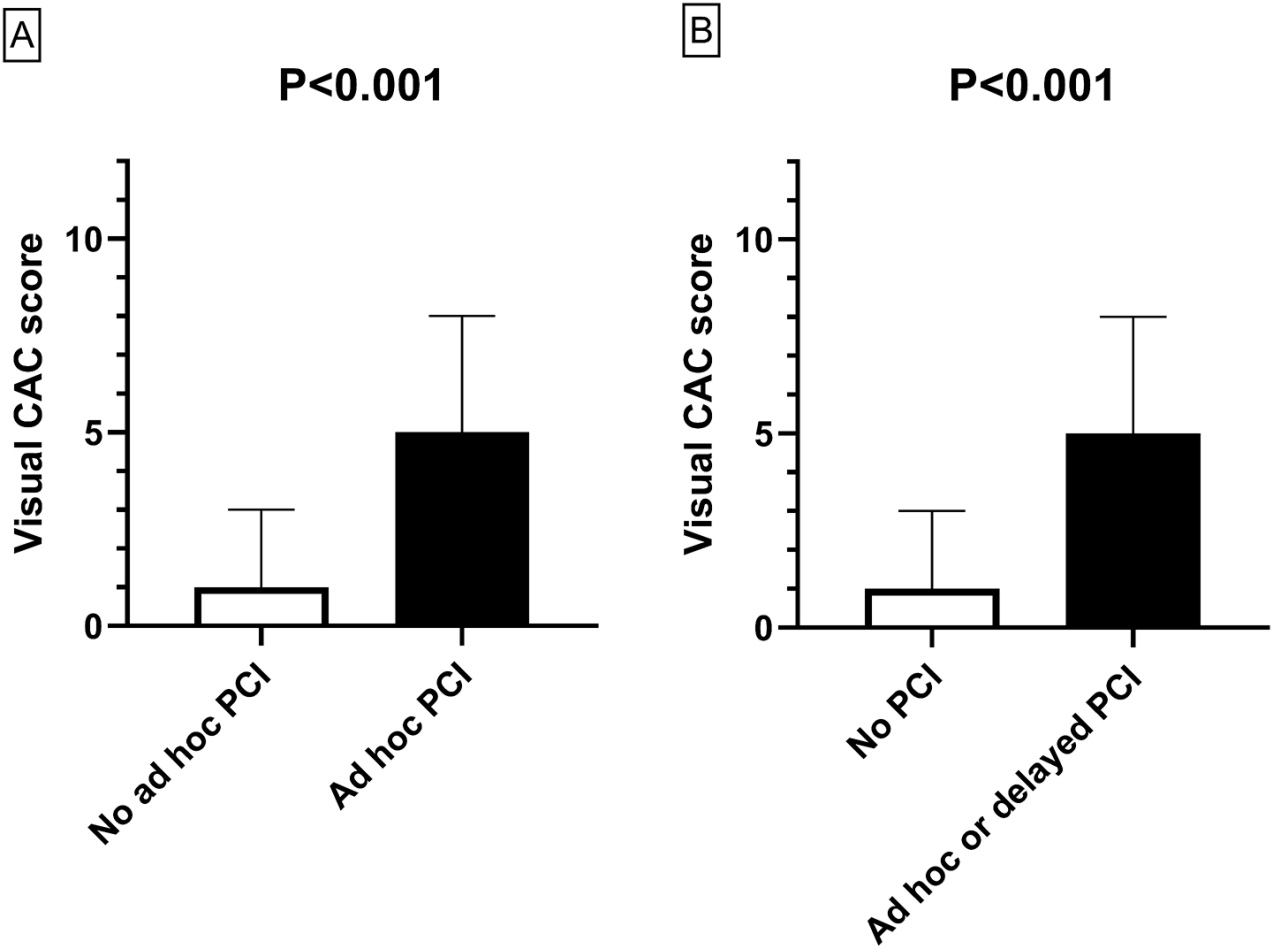
VCAC (visual coronary artery calcification) score according to percutaneous coronary intervention (ad hoc, panel **A**; ad hoc or delayed, panel **B**). Data are median and interquartile range, *P* value using Mann-Whitney test for panels **A** and **B**

The median [IQR] VCAC score increased gradually with the severity of the coronary artery disease (CAD) observed during the coronary angiogram (*p* < 0.001, Jonckheere-Terpstra test): 0 [0–1] for normal coronary arteries (*N* = 42), 1 [1–3] for atherosclerosis without significant coronary artery stenosis (*N* = 34), 5 [4–6] for 1-vessel disease (*N* = 21), 6 [5–8] for 2-vessel disease (*N* = 10), and 10 [7–11] for 3-vessel disease (*N* = 6; Fig. 2A). The median [IQR] VCAC score was also higher for patients with at least one culprit coronary artery stenosis at coronary angiogram (6 [5–9], *N* = 16) than those without (1 [0–3], *N* = 97, *p* < 0.001 Mann-Whitney test; Fig. 2B) or one significant coronary artery stenosis at coronary angiogram (6 [5–8], *N* = 37) than those without (1 [0–2], *N* = 76, *p* < 0.001 Mann- Whitney test; Fig. 2C). Similar results were observed when we compared VCAC score in patients with and without ad hoc PCI (6 [5–8] (*N* = 14) vs. 1 [0–3] (*N* = 99), *p* < 0.001 Mann-Whitney test; Fig. 3A) as well as those with and without ad hoc or delayed PCI (6 [5–8] (*N* = 18) vs. 1 [0–3] (*N* = 95), *p* < 0.001 Mann- Whitney test; Fig. 3B).

### Predictive value of VCAC score to diagnose significant coronary artery stenosis

**Fig. 4:**
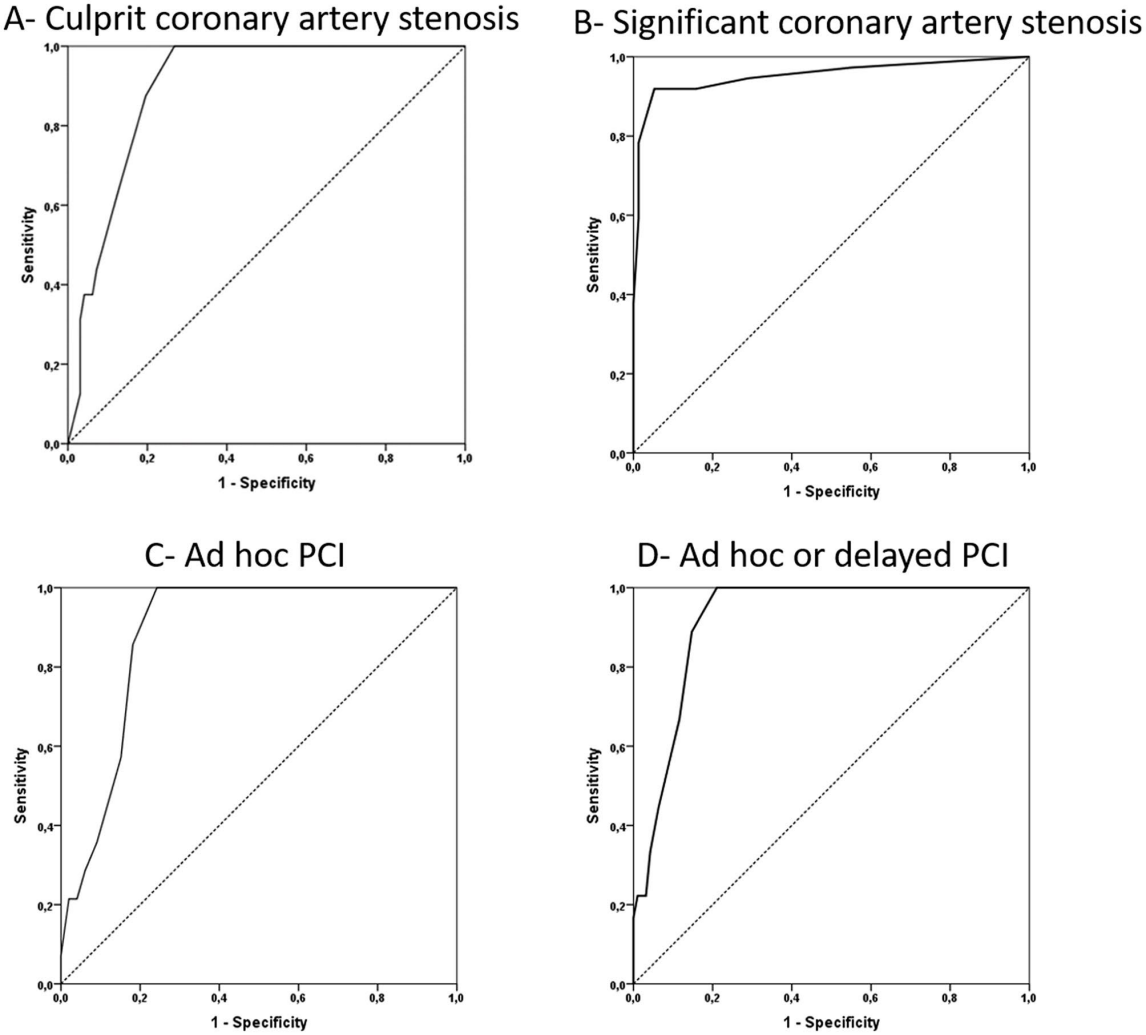
ROC curves for VCAC (visual coronary artery calcification) score to predict at least one culprit coronary artery stenosis (panel **A**), at least one significant coronary artery stenosis (panel **B**), an ad hoc PCI (panel **C**) and an ad hoc or delayed PCI (panel **D**)

The characteristics of ROC curves of VCAC score to detect at least one culprit or significant coronary artery stenosis, to perform ad hoc PCI and to perform ad hoc or delayed PCI are summarised in Table 3 and in Fig. 4 for the first physician and in Table S1 for the second physician. Overall, the performance of VCAC was excellent. The optimal VCAC score threshold was 4 for the diagnosis of at least one culprit or significant coronary artery stenosis (positive predictive value 89.5%, negative predictive value 96.0%), and 5 to predict a PCI (positive predictive value 96.7%, negative predictive value 90.4%). Subgroup analyses for asystole or ventricular fibrillation and diabetes status found also very good performance with an optimal threshold of VCAC score at 4, 5 or 6 (Table 3). A sensitivity analysis was performed after exclusion of patients who had a thorax CT performed before or after the current hospital stay (*N* = 106), and very similar results and threshold were found (Table 3). In addition, the thresholds of VCAC for a negative predictive value of 100% are presented for the different endpoints in Table 4. The threshold was < 3 for significant coronary artery stenosis, and < 4 for the other endpoints. Of note, other variables were also tested to predict coronary artery stenosis or PCI. Only age had a significant AUC for the four endpoints but with a lower performance than VCAC (Supplementary data, Table S2).

**Table 3:** Performance characteristics of VCAC score to predict significant coronary artery disease or percutaneous coronary intervention

Subgroups	AUC [95% CI]	*p*	Optimal value	Specificity (%)	Sensitivity (%)
***Performance of VCAC score to detect at least one significant coronary artery stenosis***					
Total population (*N* = 113)	0.953 [0.904-1.000]	< 0.001	≥ 4	94.7	91.9
Ventricular fibrillation (*N* = 46)	0.910 [0.807-1.000]	< 0.001	≥ 4	93.3	82.4
Asystole (*N* = 67)	0.989 [0.970-1.000]	< 0.001	≥ 4	95.7	100.0
CT during hospital stay (*N* = 106)	0.950 [0.897-1.000]	< 0.001	≥ 4	94.4	91.2
Diabetes (*N* = 27)	0.994 [0.976-1.000]	< 0.001	≥ 4	93.7	100.0
No diabetes (*N* = 86)	0.969 [0.934-1.000]	< 0.001	≥ 4	93.7	95.7
***Performance of VCAC score to detect a culprit coronary artery stenosis***					
Total population (*N* = 113)	0.903 [0847-0.958]	< 0.001	≥ 5	83.5	87.5
Ventricular fibrillation (*N* = 46)	0.907 [0.817–0.997]	< 0.001	≥ 5	89.2	88.9
Asystole (*N* = 67)	0.931 [0.855-1.000]	< 0.001	≥ 4	75.0	100
CT during hospital stay (*N* = 106)	0.914 [0.859–0.968]	< 0.001	≥ 5	84.6	86.7
Diabetes (*N* = 27)	0.880 [0.732-1.000]	0.017	≥ 6	87.0	75.0
No diabetes (*N* = 86)	0.916 [0.858–0.975]	< 0.001	≥ 4	86.5	91.7
***Performance of VCAC score to predict***ad hoc***PCI***					
Total population (*N* = 113)	0.886 [0.823–0.948]	< 0.001	≥ 5	81.8	85.7
Ventricular fibrillation (*N* = 46)	0.914 [0.830–0.999]	< 0.001	≥ 5	89.2	88.9
Asystole (*N* = 67)	0.898 [0.791-1.000]	0.003	≥ 4	72.6	100.0
CT during hospital stay (*N* = 106)	0.895 [0.833–0.957]	< 0.001	≥ 5	82.8	84.6
Diabetes (*N* = 27)	0.880 [0.732-1.000]	0.017	≥ 6	87.0	75.0
No diabetes (*N* = 86)	0.891 [0.822–0.960]	< 0.001	≥ 5	84.2	90.0
***Performance of VCAC score to predict***ad hoc***or delayed PCI***					
total population (*N* = 113)	0.921 [0.872–0.970]	< 0.001	≥ 5	85.3	88.9
Ventricular fibrillation (*N* = 46)	0.966 [0.916-1.000]	< 0.001	≥ 5	94.3	90.9
Asystole (*N* = 67)	0.907 [0.827–0.988]	< 0.001	≥ 4	75.0	100.0
CT during hospital stay (*N* = 106)	0.911 [0.856–0.966]	< 0.001	≥ 5	82.8	84.6
Diabetes (*N* = 27)	0.895 [0.769-1.000]	0.007	≥ 6	90.9	80.0
No diabetes (*N* = 86)	0.929 [0.876–0.982]	< 0.001	≥ 5	87.7	92.3

AUC, area under ROC curve; VCAC, visual coronary artery calcification; PCI, percutaneous coronary intervention; CT, computed tomography

**Table 4:** Threshold of VCAC score to exclude coronary artery disease or percutaneous coronary intervention in the total population (*N* = 113)

Objectives	Threshold of VCAC score	Specificity (%)	Sensitivity (%)	Negative predictive value (%)	Positive predictive value (%)
Culprit coronary artery stenosis	< 4	77.3	100	100	33.6
Significant coronary artery stenosis	< 3	80.9	100	100	86.8
*Ad hoc* PCI	< 4	75.8	100	100	36.8
*Ad hoc* or delayed PCI	< 4	78.9	100	100	47.4

VCAC, visual coronary artery calcification; PCI, percutaneous coronary intervention

## Discussion

**Fig. 5:**
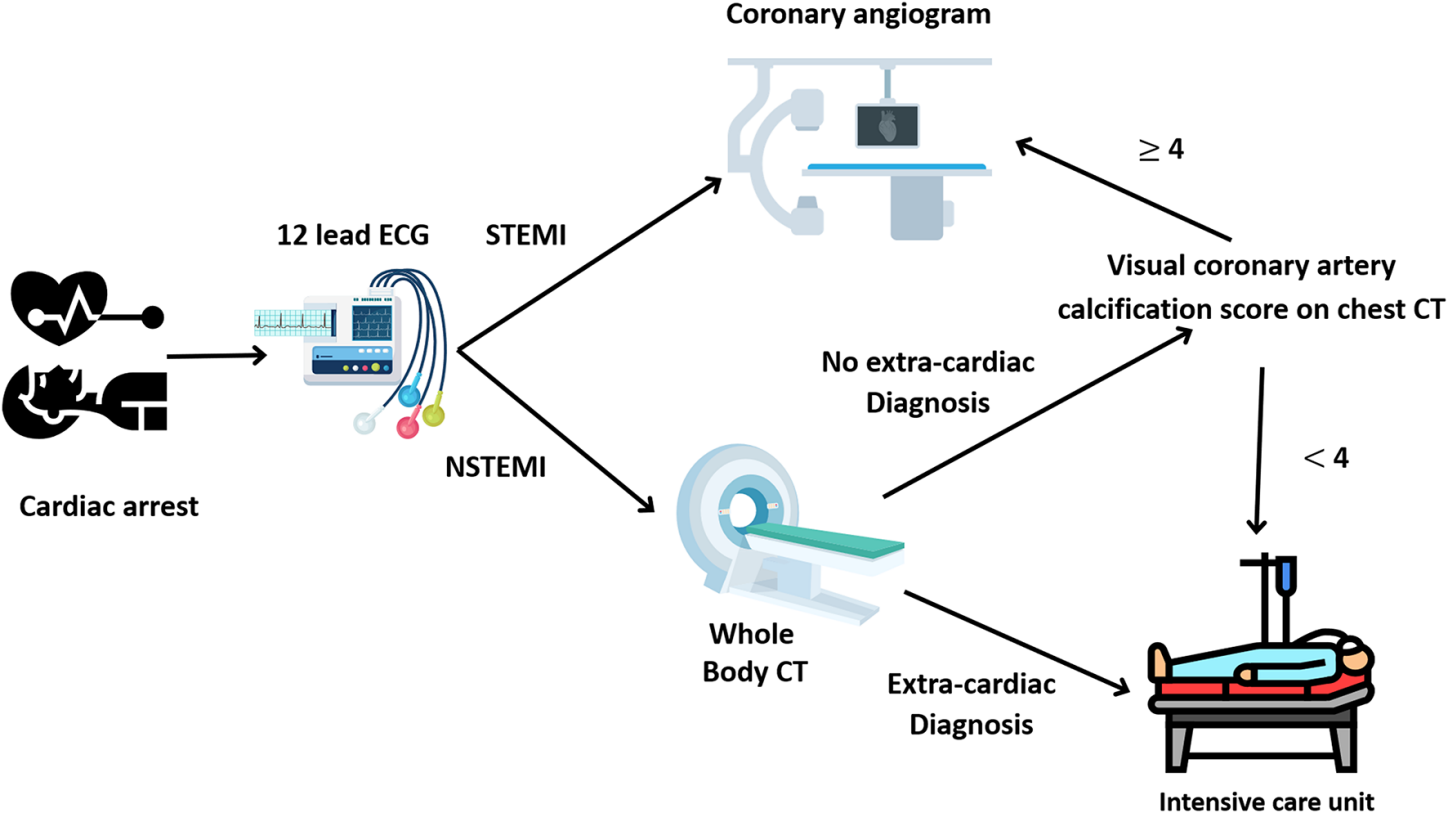
Practical algorithm

The results of this retrospective cohort indicate that VCAC score measured in a routine chest CT is a strong predictor of culprit or significant coronary artery stenosis and PCI in patients resuscitated from cardiac arrest without STEMI and without history of CAD. Whole body CT scans are widely performed at initial management of patients presenting with cardiac arrest without STEMI to diagnose the possible causes of the cardiac arrest (dissecting aortic aneurysm, cardiac tamponade, intracranial haemorrhage, pulmonary embolism, pleural effusion, pneumothorax) and also its consequences (hypoxic brain injury) [12]. As demonstrated in the present study, VCAC may be retrospectively performed by physicians without dedicated software and better select patients who may benefit from coronary angiogram and PCI if appropriate than in current practice. We propose a practical algorithm in Fig. 5 with a threshold of VCAC at 4 to optimise sensitivity to detect culprit coronary artery lesion that may benefit from PCI (negative predictive value 100%).

A threshold of VCAC at 4 was the optimal value observed to predict a culprit or significant coronary artery stenosis or PCI. This cut-off indicated the presence of diffuse VCAC at least in 2 major coronary arteries among left main, left anterior descending, circumflex and right coronary artery. We decided to retain in the analysis all patients having a CT scan one year before or after coronary angiogram because CAC score varies only marginally during this period. For instance, MESA reported results of CAC progression in 5756 participants with a mean of 2.4 years between 2 CT scans. CAC scores increased by about 20–25% per year, and about 20% of subjects with CAC = 0 progressed to CAC > 0 within 4 to 5 years [13]. Nevertheless, we performed a sensitivity analysis that included only patients having their thorax CT scan during the hospital stay and found similar results. We also tested other variables to predict coronary artery stenosis or PCI including traditional cardiovascular risk factors, shockable arrest rhythms, abnormal ECG after resuscitation, left ventricular systolic function at admission, haemodynamic status, troponin peak etc. In addition to VCAC score, the independent predictors were only hypertension and ventricular fibrillation at first medical contact. Previous studies have also explored these predictors and found that ventricular fibrillation, troponin, ECG criteria or combination with echocardiographic may be helpful to select patients for immediate coronary angiography [14–19]. However, sensitivity and specificity of these variables were lower than VCAC in our study (one or both criteria < 70% vs. >85%).

Four randomised clinical trials (TOMAHAWK, COACT, EMERGE, and PEARL) included patients presenting cardiac arrest with non-STEMI, but failed to demonstrate a better prognosis for survival in the subgroup with immediate coronary angiogram strategy [6, 7, 20, 21]. Despite that this strategy does not seem beneficial in all patients presenting with non-STEMI cardiac arrest, the results of the present study open the way for a better selection of patients with a higher frequency of CAD and culprit lesion that may potentially increase the benefit of coronary angiogram performed after CT scan. It is of note that, although a relatively low number of patients were included in the present study, the characteristics of these are close to those of other studies that included patients with non-STEMI cardiac arrest. However, we excluded patients with previous CAD to avoid misinterpreting VCAC in the presence of coronary stenting or coronary artery bypass grafting, which may explain a lower rate of culprit lesions observed in the present study. For instance, we observed significant coronary artery stenoses during coronary angiogram in 37 (32.7%) patients and Ad hoc PCI was performed in 14 (12.4%) for culprit lesion; in comparison, in the TOMAHAWK, COACT, EMERGE and PEARL trials, PCI was performed in 25 to 40%, with a culprit lesion observed between 15 and 38% [6,7,20,21].

From a practical point of view, the data presented herein suggest that patients resuscitated from cardiac arrest without STEMI and with a VCAC < 4 would probably not benefit from a coronary angiogram. Conversely, those with VCAC ≥ 4 may have a higher prevalence of culprit coronary artery stenosis and would possibly benefit from being explored by coronary angiogram. This could have important consequences for hospitals without cardiac catheterisation laboratory to avoid an unnecessary transfer to another hospital. Moreover, it may decrease the risk of bleeding or acute kidney injury in this population because coronary angiogram requires administration of both anticoagulant and contrast dye [22]. Finally, immediate coronary angiogram may delay the treatment of causes of cardiac arrest other than acute coronary syndrome requiring whole body CT and neurological protection including early targeted temperature management that is beneficial for neurological outcome [23]. All these hypotheses must be tested in appropriate dedicated studies. Coronary CT is also another imaging technique which may be useful in this situation, but it may be not available in emergency in many hospitals.

### Limitations

The major limitation of the present study is its retrospective and single-centre design with a limited number of participants. As the present study does not include a validation cohort, it would therefore be of value to further explore this in a future multicentre study. In addition, a selection bias cannot be excluded as an unknown number of patients with cardiac arrest without ST-segment elevation myocardial infarction were admitted to the intensive care unit and did not have coronary angiogram in different clinical context. It should also be noted that the VCAC score is likely to have a poor performance in young patients with coronary artery dissection or intramural haematoma that occurs most frequently in patients without coronary artery calcifications. However, we cannot further comment on this, because we did not observe any such case in the study population. Despite these limitations, VCAC required a minimal training; the resident cardiologist required only 20 chest CT interpretations for adequate inter-reader reproducibility to be achieved (during the training phase calcifications of aortic valve and mitral annulus or pericardium were notably misinterpreted as coronary artery calcifications). In addition, our experience indicates that the interpretation of VCAC is rapidly achieved, although it does vary from 2 to 5 min according to the extent of coronary artery calcifications.

## Conclusions

The present study found that a non-dedicated CT thorax may be useful to measure VCAC and if this latter is scored ≥ 4 it allows physician to better select patients resuscitated from cardiac arrest with non-STEMI and without history of CAD who may benefit from an emergency coronary angiogram to detect a significant or culprit lesion and had PCI if appropriate.

## Electronic supplementary material

Below is the link to the electronic supplementary material.

Supplementary Material 1

Supplementary Material 2

## Data Availability

The datasets used and/or analysed during the current study are available from the corresponding author on reasonable request.
