# Supplementary material for: Visual coronary artery calcification score to predict significant coronary artery stenosis in patients presenting with cardiac arrest without ST-segment elevation myocardial infarction
Source: Ann Intensive Care. 2025 Apr 7;15:50. doi: 10.1186/s13613-025-01423-5 (PMC11977084; doi:10.1186/s13613-025-01423-5)
Supplement: Supplementary file 1 — Supplementary Material 1 [file 13613_2025_1423_MOESM1_ESM.docx]

Abbreviation lists

AUC: area under ROC curve

BMI: body mass index

CT: computed tomography

eGFR: estimated glomerular filtration rate

IQR: interquartile range

LVEF: left ventricular ejection fraction

PCI: percutaneous coronary intervention

ROC: receiver-operating characteristic

SCD: sudden cardiac death

STEMI: ST-segment elevation myocardial infarction

ULN: upper limit of normal

VCAC: visual coronary artery calcification
