# Supplementary material for: Visual coronary artery calcification score to predict significant coronary artery stenosis in patients presenting with cardiac arrest without ST-segment elevation myocardial infarction
Source: Ann Intensive Care. 2025 Apr 7;15:50. doi: 10.1186/s13613-025-01423-5 (PMC11977084; doi:10.1186/s13613-025-01423-5)
Supplement: Supplementary file 2 — Supplementary Material 2 [file 13613_2025_1423_MOESM2_ESM.docx]

Supplementary data

Table S1. Performance characteristics of VCAC score interpreted by the second reader to predict significant coronary artery disease or percutaneous coronary intervention

| Subgroups | AUC [95% CI] | p | Optimal value | Specificity | Sensitivity | |
| --- | --- | --- | --- | --- | --- | --- |
| ***Performance of VCAC score to detect a culprit coronary artery stenosis*** | | | | | | |
| Total population (N=113) | 0.899 [0.841-0.956] | <0.001 | ≥4 | 80.4 | 87.5 |  |
| ***Performance of VCAC score to detect at least one significant coronary artery stenosis*** | | | | | | |
| Total population (N=113) | 0.920 [0.850-0.990] | <0.001 | ≥4 | 89.5 | 91.9 | |
| ***Performance of VCAC score to predict* ad hoc *PCI*** | | | | | | |
| Total population (N=113) | 0.874 [0.809-0.939] | <0.001 | ≥5 | 78.8 | 85.7 | |
| ***Performance of VCAC score to predict* ad hoc *or delayed PCI*** | | | | | | |
| total population (N=113) | 0.909 [0.856-0.962] | <0.001 | ≥5 | 82.1 | 88.9 | |

AUC, area under ROC curve; VCAC, visual coronary artery calcification; PCI, percutaneous coronary intervention; CT, computed tomography

Table S2. Performance characteristics of VCAC score and other variables to predict significant coronary artery disease or percutaneous coronary intervention

| Subgroups | AUC [95% CI] | p |
| --- | --- | --- |
| ***Performance of VCAC score to detect a culprit coronary artery stenosis*** | | |
| VCAC score | 0.903 [0.847-0.958] | <0.001 |
| Age | 0.718 [0.602-0.835] | 0.005 |
| Gender (male) | 0.467 [0.312-0.621] | 0.671 |
| Diabetes (yes) | 0.506 [0.352-0.660] | 0.934 |
| Current smoking (yes) | 0.418 [0.278-0.558] | 0.296 |
| Hypertension (yes) | 0.622 [0.473-0.770] | 0.120 |
| Dyslipidaemia (yes) | 0.590 [0.430-0.749] | 0.252 |
| Peripheral artery disease (yes) | 0.500 [0.347-0.654] | 0.997 |
| Ventricular fibrillation (yes) | 0.591 [0.439-0.742] | 0.247 |
| Left bundle branch block (yes) | 0.516 [0.360-0.672] | 0.837 |
| Right bundle branch block (yes) | 0.516 [0.361-0.672] | 0.834 |
| eGFR (1mL/min) | 0.643 [0.495-0.792] | 0.067 |
| Troponin at admission normalised to ULN | 0.577 [0.450-0.704] | 0.326 |
| Peak troponin normalised to ULN | 0.663 [0.527-0.798] | 0.038 |
| LVEF (-%) | 0.603 [0.470-0.735] | 0.193 |
| ***Performance of VCAC score to detect at least one significant coronary artery stenosis*** | | |
| VCAC score | 0.953 [0.904-1.000] | <0.001 |
| Age | 0.748 [0655-841] | <0.001 |
| Gender (male) | 0.507 [0.388-0.627] | 0.905 |
| Diabetes (yes) | 0.553 [0.432-0.674] | 0.062 |
| Current smoking (yes) | 0.415 [0.305-0.526] | 0.154 |
| Hypertension (yes) | 0.634 [0.518-0.750] | 0.028 |
| Dyslipidaemia (yes) | 0.545 [0.423-0.666] | 0.463 |
| Peripheral artery disease (yes) | 0.524 [0.403-0.646] | 0.691 |
| Ventricular fibrillation (yes) | 0.545 [0.425-0.665] | 0.459 |
| Left bundle branch block (yes) | 0.489 [0.370-0.608] | 0.859 |
| Right bundle branch block (yes) | 0.520 [0.400-0.641] | 0.737 |
| eGFR (mL/min) | 0.528 [0.403-0.652] | 0.650 |
| Troponin at admission normalised to ULN | 0.423 [0.311-0.535] | 0.201 |
| Peak troponin normalised to ULN | 0.525 [0.407-0.644] | 0.675 |
| LVEF (-%) | 0.551 [0.436-0.667] | 0.400 |
| ***Performance of VCAC score to predict* ad hoc *PCI*** | | |
| VCAC score | 0.886 [0.823-0.948] | <0.001 |
| Age | 0.738 [0.839-0.959] | <0.001 |
| Gender (male) | 0.432 [0.269-0.595] | 0.410 |
| Diabetes (yes) | 0.532 [0.365-0.698] | 0.703 |
| Current smoking (yes) | 0.430 [0.279-0.581] | 0.398 |
| Hypertension (yes) | 0.627 [0.470-0.784] | 0.128 |
| Dyslipidaemia (yes) | 0.584 [0.414-0.754] | 0.313 |
| Peripheral artery disease (yes) | 0.508 [0.343-0.673] | 0.924 |
| Ventricular fibrillation (yes) | 0.627 [0.470-0.784] | 0.128 |
| Left bundle branch block (yes) | 0.521 [0.354-0.688] | 0.797 |
| Right bundle branch block (yes) | 0.576 [0.405-0.748] | 0.361 |
| eGFR (mL/min) | 0.619 [0.460-0.779] | 0.152 |
| Troponin at admission normalised to ULN | 0.519 [0.382-0.657] | 0.816 |
| Peak troponin normalised to ULN | 0.628 [0.475-0.781] | 0.124 |
| LVEF (-%) | 0.654 [0.527-0.781] | 0.065 |
| ***Performance of VCAC score to predict* ad hoc *or delayed PCI*** | | |
| VCAC score | 0.921 [0.872-0.970] | <0.001 |
| Age | 0.755 [0.642-0.868] | 0.001 |
| Gender (male) | 0.509 [0.362-0.656] | 0.904 |
| Diabetes (yes) | 0.528 [0.379-0.678] | 0.705 |
| Current smoking (yes) | 0.441 [0.303-0.580] | 0.430 |
| Hypertension (yes) | 0.647 [0.508-0.787] | 0.050 |
| Dyslipidaemia (yes) | 0.574 [0.421-0.726] | 0.327 |
| Peripheral artery disease (yes) | 0.532 [0.380-0.684] | 0.667 |
| Ventricular fibrillation (yes) | 0.614 [0.470-0.757] | 0.130 |
| Left bundle branch block (yes) | 0.503 [0.355-0.651] | 0.966 |
| Right bundle branch block (yes) | 0.575 [0.421-0.729] | 0.319 |
| eGFR (mL/min) | 0.576 [0.426-0.726] | 0.311 |
| Troponin at admission normalised to ULN | 0.462 [0.327-0.597] | 0.287 |
| Peak troponin normalised to ULN | 0.580 [0.440-0.720] | 0.287 |
| LVEF (-%) | 0.583 [0.454-0.712] | 0.270 |

Figure S1. VCAC scoring for the left anterior descending artery: panel A = 0, panel B = 1, panel C = 2 and panel D = 3. Red arrows show the absence or presence of calcifications on the left anterior descending artery.


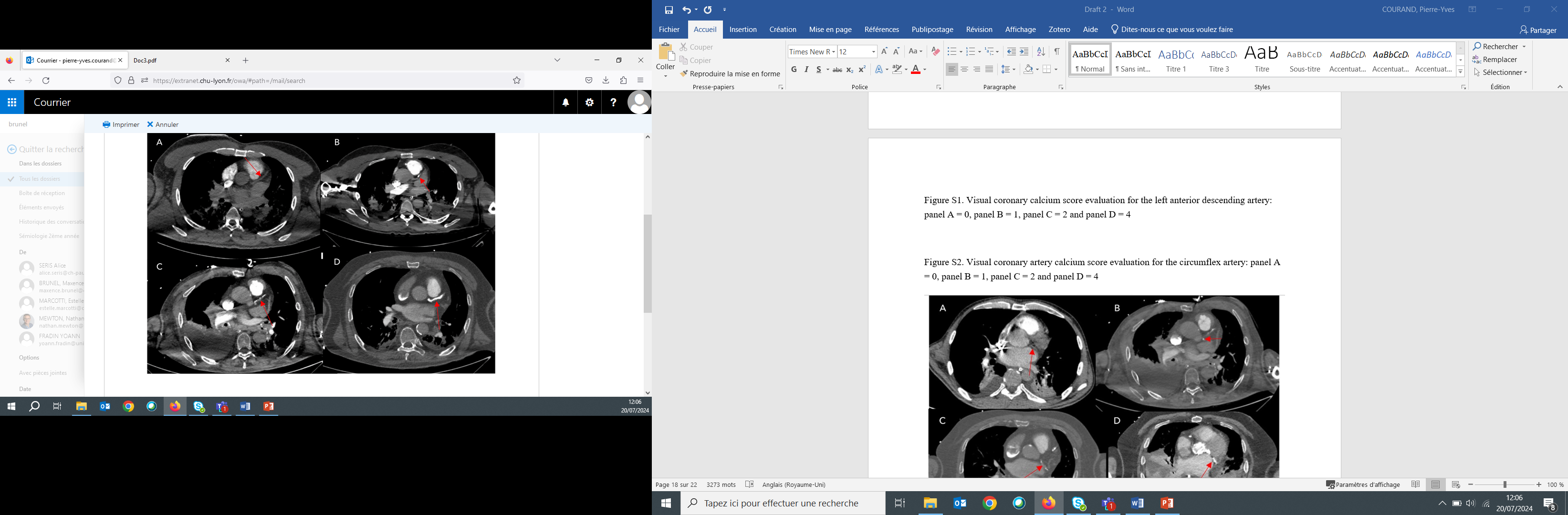


Figure S2. VCAC scoring for the circumflex artery: panel A = 0, panel B = 1, panel C = 2 and panel D = 3. Red arrows show the absence or presence of calcifications on the left circumflex artery.


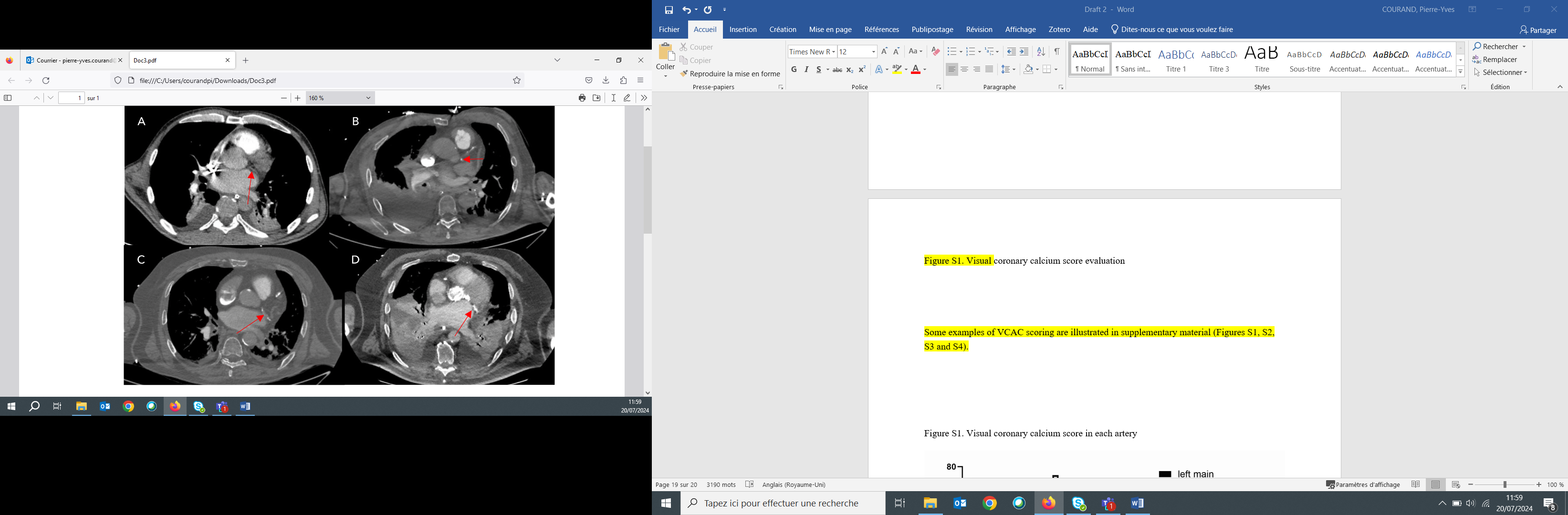


Figure S3. VCAC scoring for the right coronary artery: panel A = 0, panel B = 1, panel C = 2 and panel D = 3. Red arrows show the absence or presence of calcifications on the right coronary artery.


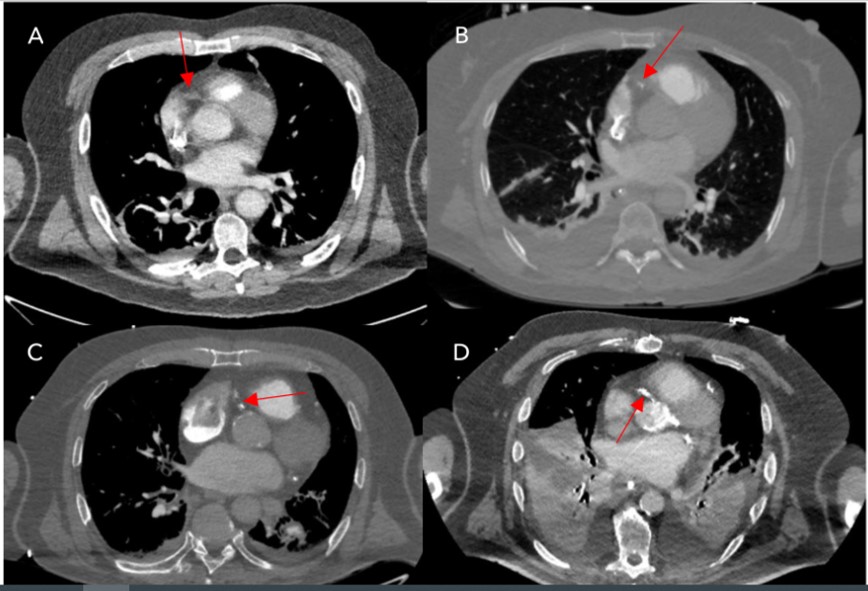


Figure S4. VCAC score in each artery


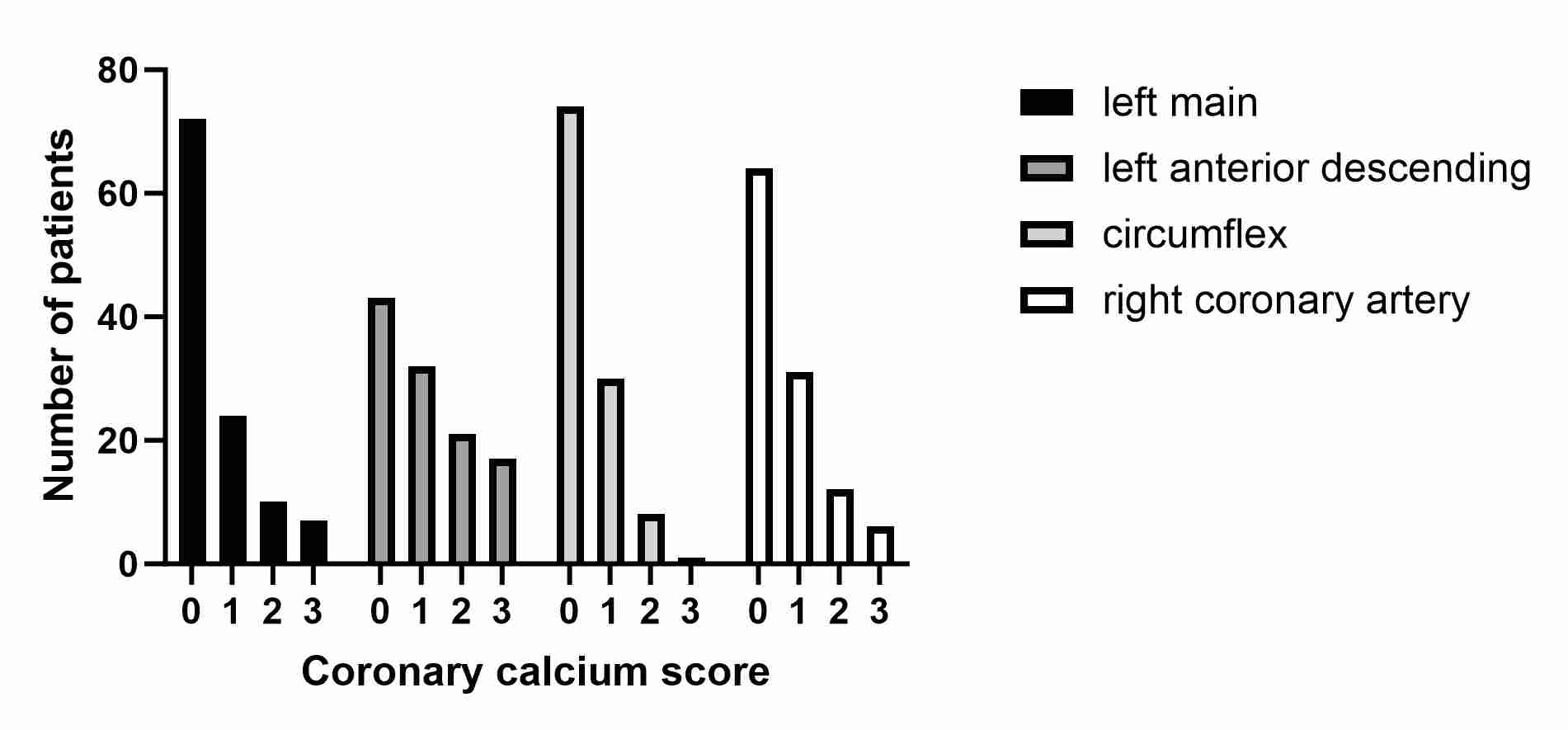


Figure S5. Total VCAC score
